# Applications for open access normalized synthesis in metastatic prostate cancer trials

**DOI:** 10.3389/frai.2022.984836

**Published:** 2022-09-12

**Authors:** Thomas Luechtefeld, Thomas Bozada, Rahul Goel, Lin Wang, Channing J. Paller

**Affiliations:** ^1^Insilica LLC, Bethesda, MD, United States; ^2^Independent Researcher, San Francisco, CA, United States; ^3^Department of Epidemiology, Johns Hopkins University Bloomberg School of Public Health, Baltimore, MD, United States; ^4^The Sidney Kimmel Comprehensive Cancer Center, Johns Hopkins University School of Medicine, Baltimore, MD, United States

**Keywords:** data curation, prostate cancer, biomarker, text mining, natural language processing, machine learning

## Abstract

Recent metastatic castration-resistant prostate cancer (mCRPC) clinical trials have integrated homologous recombination and DNA repair deficiency (HRD/DRD) biomarkers into eligibility criteria and secondary objectives. These trials led to the approval of some PARP inhibitors for mCRPC with HRD/DRD indications. Unfortunately, biomarker-trial outcome data is only discovered by reviewing publications, a process that is error-prone, time-consuming, and laborious. While prostate cancer researchers have written systematic evidence reviews (SERs) on this topic, given the time involved from the last search to publication, an SER is often outdated even before publication. The difficulty in reusing previous review data has resulted in multiple reviews of the same trials. Thus, it will be useful to create a normalized evidence base from recently published/presented biomarker-trial outcome data that one can quickly update. We present a new approach to semi-automating normalized, open-access data tables from published clinical trials of metastatic prostate cancer using a data curation and SER platform. Clinicaltrials.gov and Pubmed.gov were used to collect mCRPC clinical trial publications with HRD/DRD biomarkers. We extracted data from 13 publications covering ten trials that started before 22nd Apr 2021. We extracted 585 hazard ratios, response rates, duration metrics, and 543 adverse events. Across 334 patients, we also extracted 8,180 patient-level survival and biomarker values. Data tables were populated with survival metrics, raw patient data, eligibility criteria, adverse events, and timelines. A repeated strong association between HRD and improved PARP inhibitor response was observed. Several use cases for the extracted data are demonstrated *via* analyses of trial methods, comparison of treatment hazard ratios, and association of treatments with adverse events. Machine learning models are also built on combined and normalized patient data to demonstrate automated discovery of therapy/biomarker relationships. Overall, we demonstrate the value of systematically extracted and normalized data. We have also made our code open-source with simple instructions on updating the analyses as new data becomes available, which anyone can use even with limited programming knowledge. Finally, while we present a novel method of SER for mCRPC trials, one can also implement such semi-automated methods in other clinical trial domains to advance precision medicine.

## Introduction

Interventional biomarker clinical trials report associations between biomarkers, interventions, and patient outcomes. Biomarker-based outcomes and eligibility criteria are now common in cancer clinical trials. Most of the biomarker analyses reported for these trials are only accessible in difficult-to-access publications, with no controlled data access. Published biomarker outcomes are used in clinical guidelines, drug labels, and research. For example, the olaparib drug label references the PROfound trial data (de Bono et al., 2020; Hussain et al., 2020; FDA, 2020), and multiple publications have reviewed PARP inhibitors in mCRPC (Luo and Antonarakis, 2019; Antonarakis et al., 2020a; Jang et al., 2020). These activities involve a redundant, error-prone, time-consuming, and laborious data aggregation step.

Normalizing reported outcomes into an open-access database that can be easily updated as new data becomes available can help address this redundancy and improve the reusability of currently difficult to access biomarker outcome data. We present an approach to extracting the metastatic castration-resistant prostate cancer (mCRPC) trials data across different studies and normalizing it. The aggregated data tables and figures are also made available in an open-access format with a codebase that can be used to update them as new data becomes available. Biomarker, therapy, and outcome data were normalized into common tabular formats to analyze homologous recombination and DNA repair deficiency (HRD/DRD) biomarkers. The analyses herein can be fully reproduced in a short R script without the need for access to local files. The reviewer(s), time of review, and extracted data for each document can be looked up by readers and updated easily. We used Sysrev (Bozada et al., 2021), a web application designed to facilitate data curation and systematic evidence reviews (SER). It has an integrated machine learning (ML) platform with natural language processing (NLP) tools for searching, tagging, and extracting data from database sources. At a fundamental level, Sysrev helps to curate secondary data from biomedical research publications, and analyze them.

The potential value of the extracted and normalized data is demonstrated *via* several visualizations, which help to evaluate associations between therapies and DRD/HRD biomarkers in mCRPC trials. Computational access to these data points has the potential to accelerate precision medicine by enabling programmatic exploration of trial outcomes. This will become even more relevant in the future as results from more clinical trials get published and it will become even more difficult to search and screen for appropriate papers to include in a review, if done manually, to synthesize results across studies. Access to databases that can provide this data easily can facilitate clinical trial development and provide more transparency for guidelines, drug labels, and research.

Five analyses are done: biomarker usage counts, biomarker conditional treatment hazard ratios, biomarker response rates, adverse event comparisons, and automated discovery of therapy/biomarker relationships.

Many of these analyses focus on comparisons between the AR-directed (ARD) therapies abiraterone acetate + prednisone/enzalutamide and PARP inhibitors (PARPi) olaparib, rucaparib, veliparib, niraparib, and talazoparib. HRD specifically is widely recognized to mediate PARPi survival benefit, potentially *via* synthetic lethality (Jang et al., 2020).

Our approach demonstrates that normalizable biomarker/outcome data exist in prostate cancer clinical trial publications and identifies an opportunity to accelerate precision medicine by making this data computationally accessible. Precision oncology could benefit from efforts to collect this normalized data from clinical trials rather than re-collecting it in a systematic literature review (SLR), which is often outdated by the time it is published ((Beller et al., 2013)). We do not intend to promote SLR as the ideal solution for collecting this data. In The Future Of Annotation/Biocuration, Amos Bairoch states, “It is quite depressive to think that we are spending millions in grants [clinical trials] for people to perform experiments, produce new knowledge, hide this knowledge often in badly written text and then spend some more million trying to second guess what the authors really did and found.” (Bairoch, 2009).

## Materials and methods

### Search strategy

The first step in the process was to carry out a review of biomarkers associated with therapeutic outcomes (BAWTO), from clinical trial publications. We extracted biomarkers matching one or more HRD/DRD biomarkers in the list (BRCA1, BRCA2, ATM, FANCA, PALB2, CHEK2, BRIP1, HDAC2, ATR, MLH1, MRE11A, NBN, RAD51C, RAD51, MLH3, ERCC3, MRE11, RAD51B, BARD1, CDK12, CHEK1, FANCL, PPP2R2A, RAD51D, RAD54L) (Luechtefeld et al., 2021).

**Figure 1:**
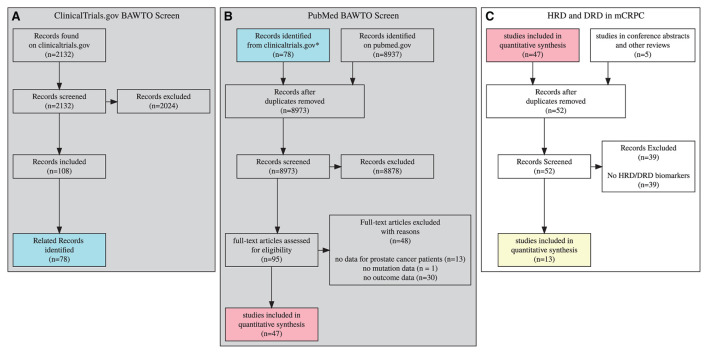
3 stage prisma diagram, gray diagrams indicate screens completed in the BAWTO project. Shared colors indicate equivalent sets. **(A)** Clinicaltrials.gov documents screened in the BAWTO project. **(B)** Publications derived from pubmed or from **(A)** are screened in the BAWTO project. **(C)** Studies screened from BAWTO and other sources for reference to HRD/DRD biomarkers.

A multi-stage Prisma diagram (Figure 1) describes the screening and search process for included documents (Page et al., 2021). Columns A and B describe the BAWTO review, which screens documents from PubMed.gov and Clinicaltrials.gov.

Briefly, the BAWTO review performs three screening steps. In each screening stage, documents were reviewed in duplicate, with conflicts resolved by a project administrator not involved in the original document review. Documents were included during each screening process when they contained three primary features:

1. A therapeutic intervention such as a drug, combination of drugs, surgery, or radiation.
2. A prostate cancer population with a well-defined mutation.
3. A measured patient outcome such as overall survival, adverse events, and progression-free survival.

In column A, the BAWTO review screened 2,132 documents from Clinicaltrials.gov matching the query for text matching “prostate”, study type “interventional,” and the overall status of “Completed” or “Active, not recruiting” as of April 22, 2021. These 2,132 documents were reviewed by five researchers with varying levels of research experience (three PhDs, BS, current university student); there were 58 conflicts which were resolved. In column B, the BAWTO review used a PubMed query for “prostate cancer” with the PubMed clinical trial filter. On April 29, 2021, this query generated 8,937 articles. The 8,937 were reviewed by the same five researchers. There were 52 conflicts which were resolved. The articles resulting from the two screens were deduplicated and overall 95 articles underwent a full text screen and initial data extraction phase, performed by four researchers (three PhDs, BS). Forty-seven articles were found to have the primary features stated above for any biomarker.

Column C in Figure 1 describes the screening process completed in this HRD/DRD biomarker trials review. Forty-seven articles were sourced from BAWTO, and five reports were collected by searching conference abstracts and identifying relevant citations from other reviews of HRD/DRD in mCRPC. The data already extracted from these documents was reviewed to identify articles with HRD/DRD biomarkers. Of the 52 resulting documents, 39 exclusions occurred due to a lack of HRD/DRD biomarkers. In total, we collected 13 documents for evidence synthesis over the course of 3 months.

### Evidence synthesis

Sysrev.com was used to extract, transform, and load data from documents into controlled “labels”. Labels are boolean, string, categorical, or tabular forms that constrain user input in order to build consistent, structured data. Data were accessed and analyzed in R using the *rsr* package (Bozada et al., 2021).

### Label design

During evidence synthesis, we extracted five primary tables from each document: Biomarker measurement, Relative population outcomes, Single population outcomes, Single population response rates, and Adverse Events. We also created an uncontrolled sixth table to contain patient-level data, including specific days to death, days to last follow-up, day of progression, biomarker status, and trial arm membership data. Future iterations of this project will attempt to control patient-level data, but the heterogeneity of patient-level data within and between publications made creating a normalized patient outcomes table too challenging for the current scope of this work.

### Biomarker measurement

A simple label was constructed to associate gene names with their use as biomarkers in the respective study. Genes were categorized as used for inclusion, used for exclusion, measured, or not measured. Genes that were both measured and used for inclusion/exclusion, were only marked as used for inclusion/exclusion.

### Relative population outcome

The “relative population outcome” table records hazard ratios as computed by univariate and multivariate Cox proportional hazards models for overall survival, prostate specific antigen (PSA) progression-free survival, and radiographic progression-free survival (Cox, 1972). The table captures treatment and biomarker descriptors for an experimental population and a control population. In total, 105 hazard ratios were extracted, with 47 hazard ratios comparing treatment outcomes for populations with the same biomarker status, and 58 hazard ratios comparing outcomes between populations with the same treatment but different biomarker statuses.

### Single population outcome

The “single population outcome” table captures overall survival and progression-free survival median duration and confidence intervals. These tables define the population by known prior treatment, biomarker status, and treatment type. Two hundred and ten single population outcomes were recorded, 78 median overall survival measurements, and 132 median progression-free survival-based measurements.

### Single population response rates

The “single population response rate” table defines a population by biomarker status, treatment, and known prior treatment. Two hundred and seventy single population response rates were recorded, including measurements of radiographic response (134), PSA response (94), circulating tumor cell reduction (10), and death (32).

### Patient-level data

Four studies associated patient identifiers with biomarker status and survival/response rate metrics. These data were often provided in heatmap form and some supplementary tables. Patient-level data were collected but have not yet been transformed into a normalized table.

### Adverse event data

A simple table associates adverse event names with grade and event counts. This table suffers from uncontrolled vocabulary, but many trials used consistent naming. Some of these data were also available on Clinicaltrials.gov, but many trials did not have AE data available on Clinicaltrials.gov.

### Clinicaltrials.gov trial protocols and metadata

Study protocols were downloaded from Clinicaltrials.gov and included with their associated publications for reference during document review. Trial metadata describing phase, eligibility criteria, design, primary and secondary objectives were accessed programmatically *via* the Tasneem et al. (2012). Clinical trial events (start, end, etc.) were manually extracted from historical Clinicaltrials.gov updates.

### Pathway databases and genomic data commons access

Normalized biomarker data can be compared to other public databases. We found homologous recombination repair (HRR) genes that were missing in the reviewed papers by accessing KEGG, wikipathways, and reactome pathway databases with the msigdbr package in R (Dolgalev, 2020). The referenced pathways included:

1. KEGG_HOMOLOGOUS_RECOMBINATION
2. WP_HOMOLOGOUS_RECOMBINATION
3. REACTOME_HDR_THROUGH_HOMOLOGOUS_ RECOMBINATION_HRR

Missing gene relevance was measured by counting occurrences in the genomic data commons prostate cancer cohort with the TCGABiolinks package (Colaprico et al., 2015) and code: “GDCquery_Maf (“PRAD”, pipelines=”muse”)”.

### Model-based recursive partitioning

Model-based recursive partitioning (MOB) was used to construct decision trees that optimally separate populations on treatment hazard ratios. MOB trees and random forests were created, using the R package MOB for recursive partitioning and the R survival package for Cox modeling (Therneau and Grambsch, 2000; Zeileis et al., 2010; Garge et al., 2013; Therneau, 2021).

All patient-level data were collected into a single table to build MOB trees. Because different publications had different methods for annotating gene alterations, we chose to mark genes as either “altered” or “normal”. This simplifies model building at the cost of losing features like zygosity, which is an opportunity for future research.

The MOB tree for patient-level data from the Chinnaiyan study (Hussain et al., 2017) was built using all the Chinnaiyan genes. Parameters included a minimum of 20 patients in leaf nodes and no Bonferroni or confidence constraints.

The MOB tree for combined Chi/Chinnaiyan data used genes (1) measured in both trials and (2) had at least 10 patients for every combination of alteration status and treatment status. This tree was restricted to 15 children in each leaf node, a maximum depth of 5, and had no Bonferroni or confidence constraints.

The MOB random forest combined TRITON2/Chi/Chinnaiyan data. In this forest, we assumed that unmeasured genes were not altered. The forest was built from 10 trees and required a minimum of 10 patients in every leaf node. Trees used binary features for BRCA1,BRCA2,ATM,TP53,PTEN,AR, and engineered binary pathway features set to true if any gene in the pathway was altered - PI3K pathway, AR associated pathway, DNA repair. A final feature, “multiple alterations,” was true if the patient had more than two alterations in any gene.

### Plotting

Base R, meta and metafor packages were used to generate confidence intervals, response rates, and forest plots (Viechtbauer, 2010; Balduzzi et al., 2019). The treemap visualization uses the plot_ly package with treemap layout (Inc. PT, 2015). Other visualizations involved custom code, ggplot, ggparty, patchwork and ggkm (Wickham and Wickham, 2007; Pedersen, 2017; Borkovec and Madin, 2019; Sachs, 2020).

## Results

### Trial characterization

Ten mCRPC clinical trials were identified that measured biomarkers for HRD or DRD (Table 1). Three trials had multiple publications, some having preliminary/final results (PROfound-1 vs. PROfound-2) and some having reports on different stages and subgroup analyses (TOPARP-A vs. TOPARP-B and TRITON2-1 and TRITON2-2).

**Table 1:** Ten mCRPC trials [0mm][8mm]measuring HRD or DRD biomarkers were included with results reported in 13 publications.

**Trial**	**Study**	**Intervention(s)**	**Assignment**	**Brief observation(s)**	**Patient**	**Study type**
NCT02987543	1. PROfound-1 2. PROfound-2	1. A1 - Olaparib 2. A2 - Enz or AAP	Randomized Parallel	1. Stratified population with HRD to AAP/Enza or Olaparib. 2. Identified strong evidence of PARPi survival benefit for HRD	None	Complete
NCT01682772	1. TOPARP-A 2. TOPARP-B	Olaparib	Single Group	1. First mCRPC specific HRD Olaparib trial. 2. Identifies strong response rates in patients with BRCA2 mutations	Limited	Complete
NCT02952534	1. TRITON2-1 2. TRITON2-2	Rucaparib	Single Group	1. Only Rucaparib (a PARPi) trial 2. Stage 1 gives BRCA1/2 associations stage 2 focuses on non BRCA HRD.	Full	Complete
NCT01576172	Hussain et al. (2018)	1. A1 - AAP 2. A2 - AAP+Veliparib	Randomized Parallel	1. ETS Fusion stratification factor 2. identifies no significant difference between arms 3. identifies HRD survival association in both arms	Full	Complete
NCT01972217	Clarke et al. (2018)	1. A1 - Olaparib + AAP 2. A2 - Placebo + AAP	Randomized Parallel	1. Precursor to PROfound trial with the same biomarkers measured 2. weak HRD - OS relationship, but stronger HRD - rPFS benefit	None	Complete
NCT02125357	Annala et al. (2018)	1. A1 - AAP 2. A2 - Enzalutamide	Randomized Crossover	1. No difference in survival between arms in any subgroup 2. Large number of tested biomarkers identified in liquid biopsy 3. Circulating Tumor DNA strong association with survival	Full	Complete
NCT01078662	Kaufman et al. (2015)	Olaparib	Single Group	1. The only trial with diseases in addition to mCRPC. 2. The first trial applying olaparib to mCRPC	None	Complete
NCT02312557	Graff et al. (2020)	Enz + Pembro	Single Group	1. The only trial involving immunotherapy - Pembrolizumab 2. Only 16 patients with baseline biopsies 3. Potentially weak psa response in patients with HRD 16% (3–50%)	None	Complete
NCT03148795	TALAPRO-1	Talazoparib	Single Group	1. Only talazoparib (a PARPi) trial. 2. Shows a strong composite response 76.1% in BRCA1/2 patients 3. Much weaker response in non BRCA1/2 HRD (0–40%)	None	Preliminary
NCT02854436	GALAHAD	Niraparib	Single Group	1. Only Niraraparib (a PARPi) trial 2. Strong composite response in BRCA1/2 (65%) 3. Weaker composite response in non BRCA1/2 HRD (31%)	None	Preliminary

Publications were collected on April 22, 2021. The left identifier is the Clinicaltrials.gov identifier for each trial. The study column provides a trial acronym (if available), followed by −1 for the first publication and a −2 for the second. The last author's name and year of publication are provided when a trial acronym was not available. The availability of patient data is broken down into none—no data available, limited—only biomarker data available, and full—both biomarker and clinical outcome data available. The study type indicates whether the collected data were a complete peer reviewed publication(s) or a preliminary conference abstract result.

Biomarkers were used as part of eligibility criteria, part of the primary or secondary objectives, and as part of the trial design. In addition to HRD/DRD, several trials measured biomarkers related to other mechanisms in prostate cancer, including androgen receptor, ETS fusions, WNT, PI3K, and Cell cycle mechanisms. However, this publication primarily focuses on the outcomes associated with HRD/DRD.

The PROfound study was the only phase 3 trial identified. All other trials were phase 2 trials. There were four multi-arm trials comparing treatments in patients with and sometimes without specific HRD mutations. Three of these trials compared a PARP inhibitor against next-generation androgen receptor (AR) directed (ARD) therapies (Abiraterone Acetate + Prednisone or Enzalutamide), which are routinely used in mCRPC treatment (Cabot et al., 2012; Fizazi et al., 2012, 2017; Beer et al., 2014; Ryan et al., 2015). The remaining trials all had single group assignments and used PARP inhibitors (Talazoparib, Niraparib, Olaparib, Rucaparib, Veliparib) and, in one case, a combination of Enzalutamide and Pembrolizumab. None of the trials used radium-223 or sipuleucel-T. None of the trials used taxane-based therapies, although several had prior treatment conditions and subgroup analysis based on taxane use.

#### AR-directed therapies AAP and enzalutamide

The most common treatment class used in five trials was one of the AR-directed (ARD) therapies Abiraterone Acetate + Prednisone (AAP) or Enzalutamide. ARD was frequently used as a control (three trials) and/or in combination with another therapy (three trials), and one trial compared AAP to Enzalutamide. ARD works by targeting the androgen signaling pathway, which is critical in mCRPC progression (Sumanasuriya and Bono, 2018). Several ARD resistance mechanisms, including AR modifications, can reduce therapeutic benefits. These mechanisms may impact differential outcomes, particularly when ARD progression is an eligibility requirement.

#### PARP inhibitors

Many of the evaluated studies used PARP inhibitors like Olaparib (four trials), Rucaparib (TRITON), Veliparib (Chinnaiyan), Talazoparib (TALAPRO-1), and Niraparib (GALAHAD). PARP enzymes have an important role in single-strand DNA repair. One of the mechanisms for the therapeutic effect of PARP enzyme inhibitors is their ability to disrupt single-strand DNA repair (Dziadkowiec et al., 2016). Disruption of ssDNA repair results in the aggregation of double-strand breaks. When dsDNA repair is simultaneously disrupted *via* homologous recombination defects, like a BRCA2 alteration, the cell has no way to recover, and synthetic lethality occurs. Synthetic lethality is the primary reason for the high prevalence of PARP inhibitors as the experimental arm or therapy in the single group studies in these trials. The hypothesis is often that patients with a BRCA2 or other HRD mutation will see greater benefit from PARP inhibitors relative to existing therapies like ARD.

#### Pembrolizumab

Pembrolizumab, immunotherapy, was tested in combination with Enzalutamide in the Moran, 2020 study (Graff et al., 2020). The resulting PSA response was low relative to other trials, even in the HRD group. Still, the authors saw other metrics to conclude that Pembrolizumab “Responses were deep and durable and did not require tumor PD-L1 expression or DNA-repair defects” (Graff et al., 2020).

Taxanes, like docetaxel and cabazitaxel, were not used as an intervention or control in any of the trials. Still, many trials had conditions or subgroup analysis based on prior treatment with taxanes.

#### Timelines

**Figure 2:**
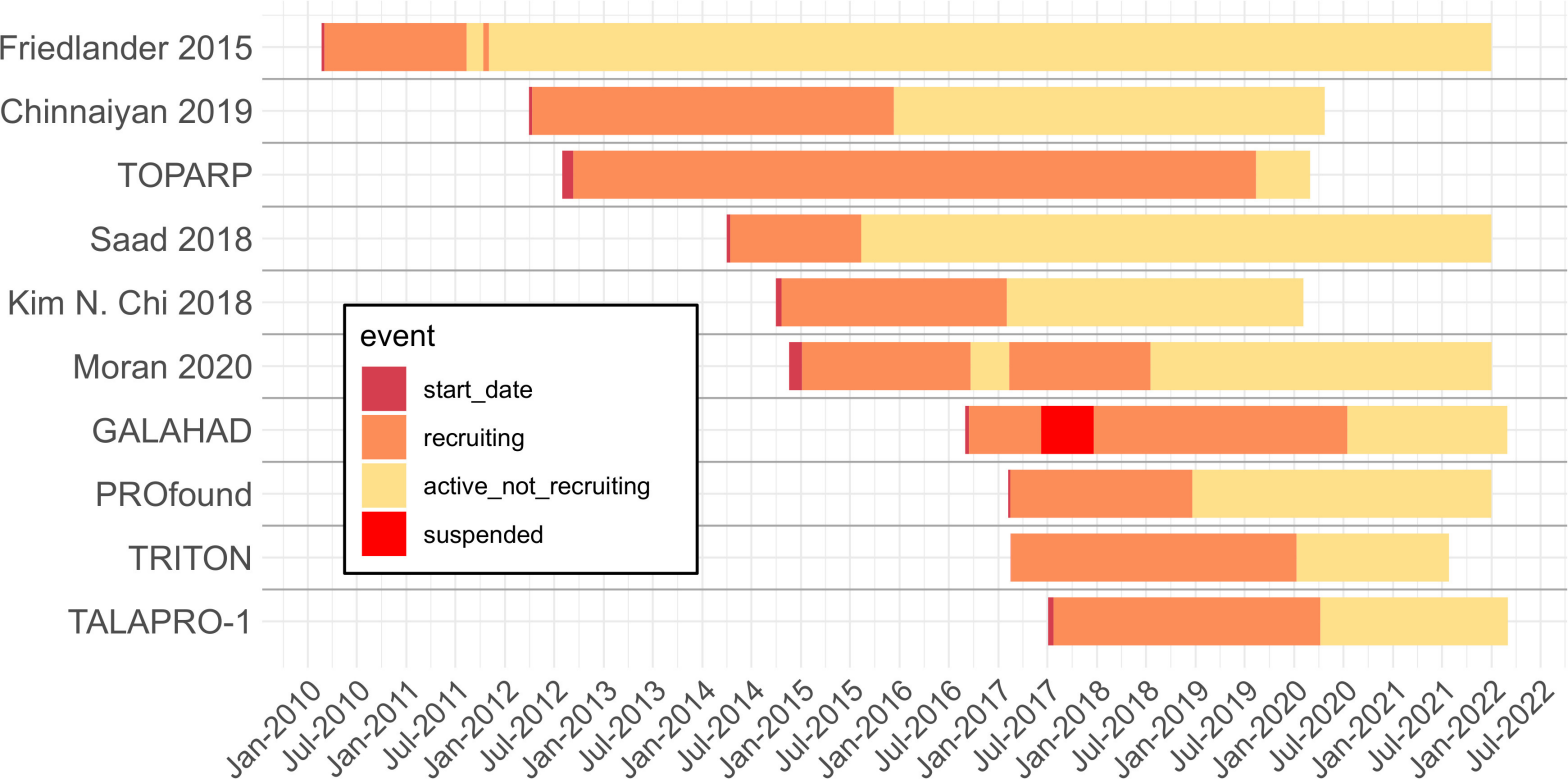
Timeline of reviewed trials with start, recruiting, active not recruiting and suspended dates extracted from Clinicaltrials.gov archive. When trials have not been completed, end dates are given by the Clinicaltrials.gov expected completion date. Dotted line is the extraction date.

Seven of the ten reviewed studies have estimated completion dates in the future, with all trials estimated to complete before 2023. All included trials have already completed recruiting. Several trials started as recently as 2017 (Figure 2). We should expect the number of publications from these trials to double or more in the next 2 years relative to the reviewed publications found and included.

### Biomarker eligibility, measurement and GDC prevalence

**Figure 3:**
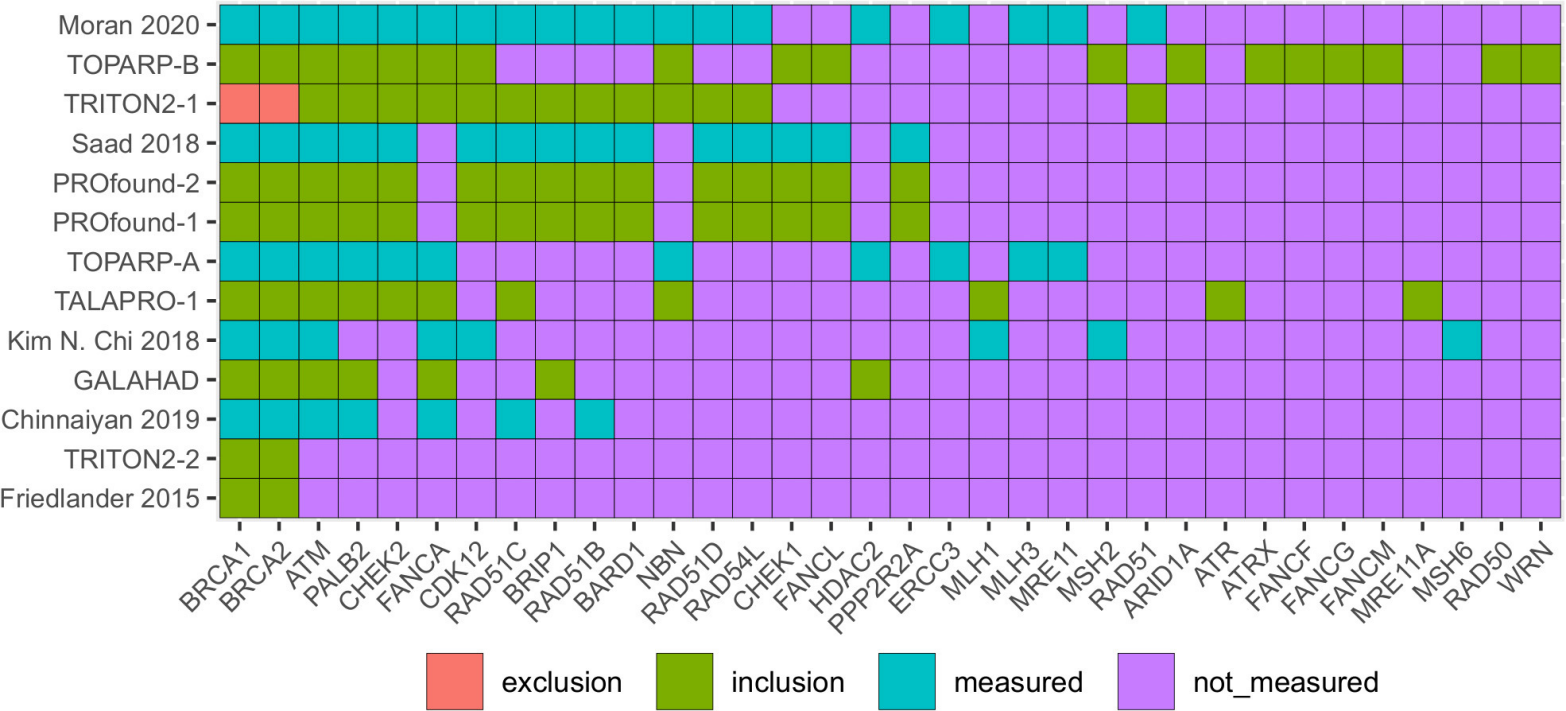
Heatmap identifying the DRD and HRD biomarkers measured in included mCRPC clinical trials. Red boxes identify biomarkers that were used to exclude patients, green used for including patients, blue biomarkers were measured but not used for eligibility. Purple biomarkers were not measured. Trials in red (on left) provide patient level data in figures or supplements.

We recorded the measurement status and use of biomarkers as eligibility criteria for each study. Figure 3 provides a heatmap with rows ordered from the study with the most HRD/DRD biomarkers to the study using the least. The columns are ordered from the biomarkers used in the most studies (BRCA1/2) to the HRD/DRD biomarkers used in the least. BRCA1/2 was used in every identified study except TRITON2-1. TRITON2-2 and Kaufman et al. (2015) were the only studies that did not measure ATM, and TRITON2-2 is a special case where ATM results were published in TRITON2-1. Some of the heterogeneity in measured genes can be explained by choices to measure HRD and DRD vs. HRD alone. For instance, the FANCA gene is not considered part of the homologous recombination repair pathway in KEGG, Wikipathway, and Reactome pathway databases.

### Unstudied HRR genes

KEGG, Wikipathway, and Reactome pathway databases identify 59 HRR genes that aren't measured in any trials. These genes may be excluded for technical reasons, low prevalence, the low likelihood of having a deleterious effect, or other reasons. By using TCGAbiolinks (Colaprico et al., 2015; Silva et al., 2016; Mounir et al., 2019) we found that these unmeasured genes do have variants in Genomic Data Commons prostate adenocarcinoma population. Variants in the measured genes occurred in 63 patients, and variants in the unmeasured genes occurred in 30 GDC prostate cancer patients with 39 Moderate and four High impact variants identified by the IMPACT algorithm (McLaren et al., 2016).

The only study that excluded participants based on a biomarker result was TRITON2-1 which focused on the relationship of non-BRCA1/2 biomarkers of HRD with rucaparib (a PARP inhibitor). TRITON2-2 complemented this analysis with a focus on populations with a BRCA1/2 mutation.

Many biomarkers were measured in addition to HRD and DRD biomarkers. Other categories of biomarkers covered androgen receptor biomarkers, cell cycle, WNT pathway, chromatin modifier, and ETS fusions.

### Treatment hazard ratios

Overall survival (OS) and progression-free survival (PFS) treatment hazard ratios (HRs) were collected from four trials and calculated from combined patient data in the Chinnaiyan, Chi, and TRITON studies.

**Figure 4:**
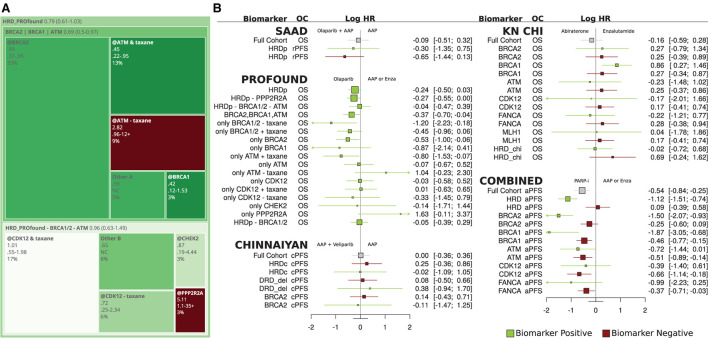
**(A)** Treemap for PROfound overall survival hazard ratios for olaparib relative to physicians' choice of AAP or Enz. **(B)** Forest plot of overall survival and progression-free survival hazard ratios for olaparib relative to AAP or physicians' choice of AAP or Enz for Saad, PROfound, Chinnaiyan, CHI, and combined trials.

In Figure 4A, PROfound OS HRs and patient counts are used to build a treemap. The overall HR is reported at the top (0.79), and subgroup hazard ratios are placed within parent groups with size proportional to prevalence and color from green to red indicating HR (Olaparib vs. AAP). The BRCA2|BRCA1|ATM group covers patients with BRCA1/2 or ATM mutations. It has five subgroups, a group with *only* BRCA2 alterations (@BRCA2), only BRCA1 (@BRCA1), prior taxane + only ATM, no prior-taxane + ATM alterations, and an “Other” group with HR found *via* pooling. These subgroups' pooled HR and patient count are equal to the BRCA2|BRCA1|ATM group.

### Prior taxane therapy and ATM

In PROfound, the overall ATM HR was 0.93 (0.53–1.75), but prior-taxane status drops to 0.45, below even the BRCA2 HR, and the absence of prior taxane raises ATM HR to 2.82. This prior-taxane/OS relation is less significant in BRCA1/2 and CDK12 subgroups (Figure 3B PROfound). CDK12 may not be a robust biomarker for HRD (Antonarakis et al., 2020b).

### Significant results and BRCA2

The PROfound study was the only study to report significant treatment HRs at the 95% confidence interval level. The Chinnaiyan, Chi, and SAAD studies did not report significant differences at the 95% level (Figure 4B). BRCA2 had the strongest reported treatment HRs of.21 (0.13–0.32) for rPFS in PROfound. Chinnaiyan did not duplicate this, perhaps due to the sample size, Veliparib specific factors, or differences in eligibility criteria.

### Combined group

The Figure 4B COMBINED group reports HR between PARPi in Chinnaiyan/TRITON2 and ARD in Chi which were chosen due to overlapping survival curves. PARPi HR was strong for the HRD group (green, row 2) relative to the full cohort and better still relative to patients who did not have HRD alterations (red).

### Response rates

**Figure 5:**
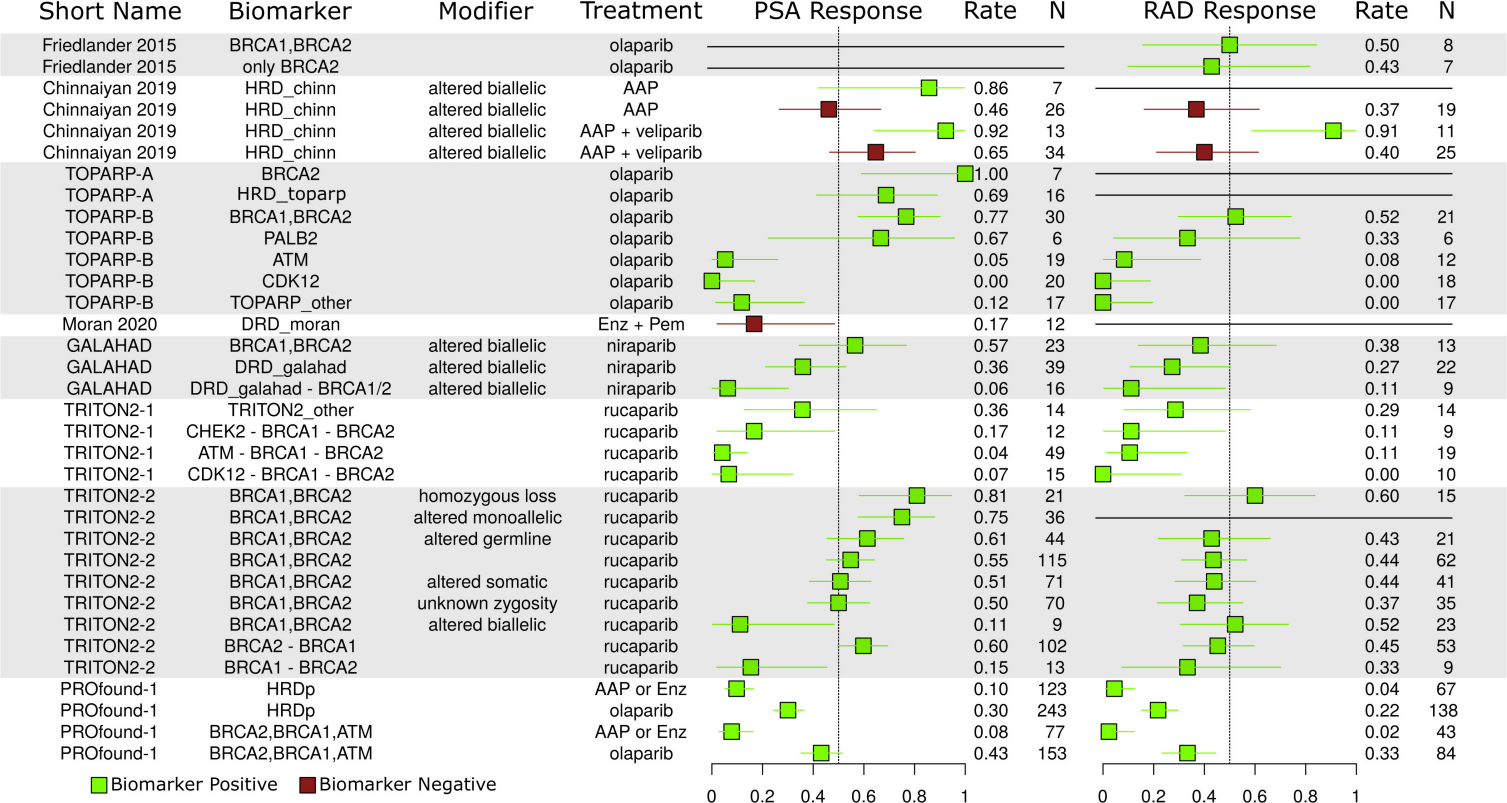
Reported PSA and radiographic (RAD) response rates. Green, population with biomarkers; Red, population w/out biomarkers.

Single population response rates were extracted from all thirteen studies. In Figure 5, simple PSA response rates (PSA drop > 50%) and radiographic (RECIST any version) response rates are given. These results are filtered to only the reported HRD/DRD response rates. Abbreviations starting with HRD or DRD correspond to groups with mutations in any HRD/DRD biomarkers recorded in the respective trial (see Figure 3).

### BRCA1/2 zygosity

Zygosity is an important consideration for DNA repair mutations. Monoallelic tumors may still have functional DNA repair pathways, and it is often hypothesized that homozygous loss/alteration is a better predictor of PARP-i response than alteration alone. In the TRITON2-2 study (2nd group from bottom), different response rates are given for BRCA1/2 based on zygosity. The homozygous loss group saw the best response rate, but the monoallelic group also saw a very strong response rate, perhaps indicating some weakness in this hypothesis.

### ATM CDK12, and non-BRCA1/2 response rate

The ATM, CDK12, and other non-BRCA1/2 groups saw poor relative response rates in the TRITON2-1, GALAHAD, and TOPARP-B studies, providing more evidence of BRCA1/2 dominance in the observed HRD/PARP-i relation.

### AAP differences

The PROfound trial ARD arm had poor response rates, possibly due to inclusion criteria requiring patients to have progressed on prior ARD therapy. The Chi study also reports this relationship and writes that HRD may prime tumors for ARD resistance (Annala et al., 2018). In the Chinnaiyan study, which did not have an ARD failure eligibility criteria, HRD positive AAP patients (green) had better aggregate response rates than those without (red).

### Adverse events

**Figure 6:**
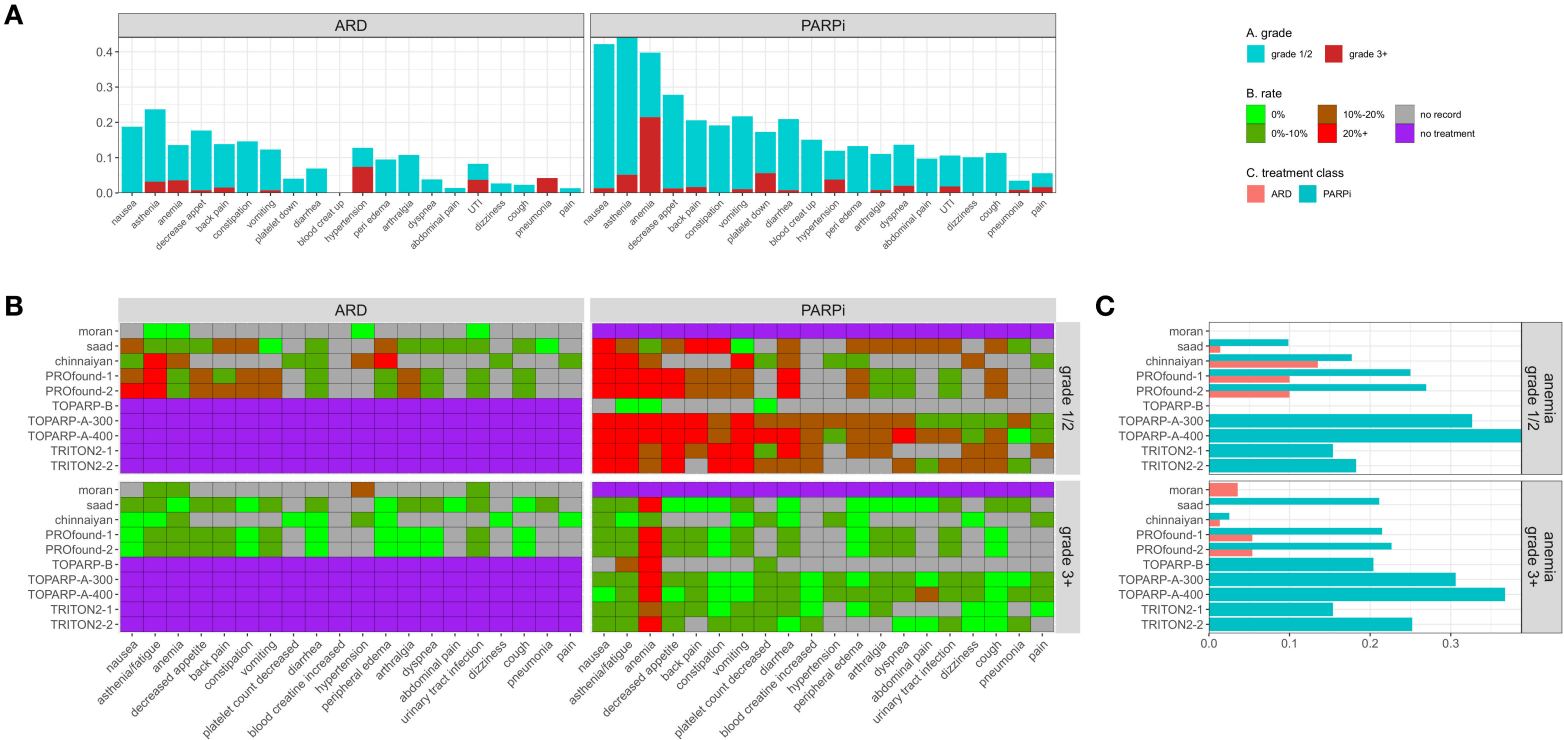
**(A)** Stacked median adverse event rates across reporting trials by grade and treatment type. **(B)** Heatmap providing reported rate of trial + adverse event pairs faceted by grade and treatment type. **(C)** Adverse event rates for anemia by trial and treatment class.

In total, 113 adverse events (AEs) types were reported in at least one study, 20 were reported in 3 or more studies and are shown in Figures 6A,B. Similar data are stored on Clinicaltrials.gov, but with less coverage (**Appendix A1**). In Figure 6A, median AE rates across grade and study are stacked and colored by grade. The median rate of serious anemia events was 20% in PARPi populations and ~20% for grade 1/2.

### ARD events

The rate of severe grade 3+ AEs was <11% in all ARD populations for all event types. Hyperglycemia in the Chinnaiyan study and anemia/asthenia in the PROfound studies were the only reported serious AEs that impacted 5+ patients at >5%. Three out of 28 patients in the Moran ARD arm had a severe hypertension event. Asthenia was the most common event in all ARD populations except Moran.

### PARP-I events

Anemia was the most common serious AE (>15%) in all PARP-i populations except the Chinnaiyan population, where only 2 (2.5%) of patients had a severe anemia event. Serious platelet count decreases and asthenia were also frequently reported at lower median rates.

AE dose dependence is seen in TOPARP-A-300 (300 mg olaparib) vs. TOPARP-A-400 (400 mg). Every serious AE affecting 2+ patients had a higher rate in the 400 mg group (except back pain). Abdominal pain, spinal cord compression, arthralgia, and hypertension affected 8%+ of the 400 mg and 0% of the 300 mg group.

### Comparison

In studies with ARD and PARPi arms, the rate of most adverse events was higher for PARPi, with significance in some studies for anemia (Figure 6C), nausea, lymphocyte decrease, pyrexia, and asthenia. The only significant risk difference in grade 3+ AE was for anemia in the PROfound-2 and Saad studies (**Appendix A2**).

### Patient-level data and simple models

**Figure 7:**
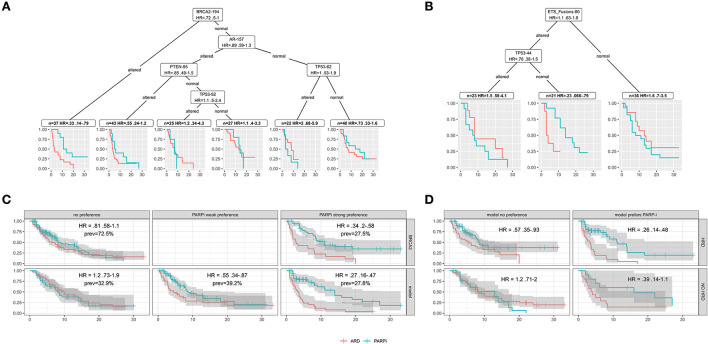
In all plots, red indicates ARD treatments and blue PARPi. **(A)** Model based recursive partitioning tree separating patients according to treatment hazard ratios (PARPi vs. ARD). **(B)** A MOB tree for patients in the Chinnaiyan study. **(C)** Survival curves evaluating BRCA2 hazard ratios for altered (top right) vs. unaltered (top left) and for patients separated into hazard ratio buckets by a ten tree MOB random forest (bottom). **(D)** Survival curves for patients with (top) and without (bottom) HRD and strong (right) or no (left) model preference for PARP-i.

In Figure 7, models are constructed from combined patient-level data from 2 to 4 trials. There are issues using combined data to build models, and here they are only meant to describe use cases for the extracted data.

### Models as research tools

In Figure 7A, combined Chinnaiyan/Chi patient data is partitioned into groups that differ the most in Cox model treatment hazard ratios. The model-based partitioning (MOB) tree (Zeileis et al., 2010) identifies BRCA2 as the strongest treatment differentiator. It prioritizes AR next, perhaps due to ARD resistance in the altered subgroup.

A tree built on all the patients in the Chinnaiyan paper (Figure 7B) identifies an ETS fusion subgroup negative for TP53 alteration with a strong PARPi preference. This subgroup provides evidence for molecular theories. ETS fusions are a factor in PARPi activity, and p53 modifications are known to impact PARP1 PARylation (Fischbach et al., 2017). Clinical researchers could use models/visualizations like these in a research tool to explore treatment hypotheses.

### Models to extend/refine drug indications

HRD can be thought of as a simple model - HRD is true if any HRR gene is altered. More complex models can add predictive power to biomarker indications. Figure 7C uses a random forest of MOB trees (Garge et al., 2013) to group patients from 4 trials by those with no, weak, or strong (top 15%) PARPi preference. In this combined cohort, BRCA2 alteration has an HR of 0.34 (upper right), and the strong PARPi preference group for the model has a lower HR of 0.21. Better stratification of patients can be useful when therapy is weighed against factors like AE risk.

Figure 7D uses the random forest to find an HRD negative subgroup that may still benefit from PARPi (lower right). The model also identifies a subgroup of HRD positive patients that may not benefit from PARPi (upper left). These examples demonstrate that complex models could extend or refine drug indications with the potential to improve trial success.

### Queryable data

While machine learning models can provide powerful aids in developing precision medicine, often what is needed are answers to simple questions. Normalized, queryable data can answer questions like:

### What was the longest reported median overall survival time in each trial?

One line of code can answer this question, and it allows us to generate ideas about the mCRPC trial's overall survival. We should not use this question to compare results. Median overall survival in months in increasing order:

1. 13.8 - BRCA1/2 negative HRD patients receiving olaparib (TOPARP-A)
2. 17.7 - BRCA1/2 altered patients receiving olaparib (TOPARP-B)
3. 22.7 - AAP + Olaparib without specified biomarker (Saad)
4. 24.8 - BRCA2 altered patients receiving olaparib (PROfound)
5. 32.3 - AAP + veliparib, without specified biomarker (Chinnaiyan)

### Which PARP-I had the lowest and highest rate of grade 3+ anemia?

A single line of code can answer this question. In increasing order, the reported rates PARPi of serious anemia:

1. 2.53% - AAP + Veliparib in Chinnaiyan
2. 15.3% - Rucaparib in TRITON2-1
3. 20.4% - TOPARP-B
4. 21.1% - AAP + Olaparib Saad
5. 22.7% - Olaparib PROfound
6. 25.2% - Rucaparib in TRITON2-2
7. 30.6% - 300 mg group TOPARP-A
8. 36.7% - 400 mg group TOPARP-A

Open access, queryable data, will help providers and clinical researchers ask and answer questions like these in real-time. These queries can be viewed at insilica.co/bawto/figure-scripts/statements.html.

## Discussion

Today, biomarker-based clinical trial outcome data are not readily accessible by oncologists, researchers, and policy makers in publicly available databases. Instead, the research, treatment, and regulatory decisions that rely on these data are limited by the need to search, read and recall literature manually. Accessibility in published literature indicates that the data are not confidential. An opportunity exists to improve data access, save time, reduce errors, and enable better therapy and research. Here, we present an approach to explore some of those possibilities in HRD/DRD biomarkers mCRPC clinical trials. Additionally, all of our work is open-source, with step-by-step instructions, and anyone with limited programming knowledge can build upon it.

The publication of trial results in closed-access journals makes it hard to access by oncologists, researchers, and policymakers. Also, the absence of computationally accessible data prevents the use of modern informatics methods on this data. When normalized, clinical trial results can be combined, which has its challenges, but can enable greater statistical power and improve our ability to understand the disease. In this review of mCRPC trials using our approach, we extracted thousands of data points from only 13 studies in a normalizable manner.

The data points analyzed in this trial are difficult and time-consuming to find. They can't be computationally accessed without human labor. They are not stored in an interoperable format so that results can be jointly analyzed. Even when the manual data extraction process is done, the results are not reusable by future researchers. In short, biomarker outcome data is not findable, accessible, interoperable, or reusable (FAIR).

In this publication, we show that for a set of 13 mCRPC HRD/DRD trial publications, outcome data for hazard ratios could be extracted, compared, and visualized, thus identifying (1) high impact biomarkers like BRCA2, (2) possible biomarker interactions with prior treatments, (3) surprising results in specific trials, like the lack of an HRD survival benefit for Veliparib in the Chinnaiyan study. The combination of semi-normalized patient data was used to build simple models to automate the discovery of interesting patient subgroups in combined data.

With access, any internet-connected machine can run this entire analysis from a simple RScript. These scripts can be viewed on insilica.co/bawto or at https://github.com/insilica/bawto.

### Models

Machine learning models like those demonstrated here can identify biomarker-outcome patterns that are too complex for humans or remain unobserved due to human bias (Maertens et al., 2020). Machine learning models can identify causal relationships, an important aspect when considering disease progression and treatment effects (Luechtefeld et al., 2020). Guidelines for using algorithms in trial designs indicate that models could be used to refine eligibility criteria or stratify patients based on model outputs from biomarker data. Guidelines like SPIRIT-AI provide suggestions and checklists to navigate the possible issues with clinical trial model use (Rivera et al., 2020).

### Limitations

Our approach was restricted to clinical trial publications and, as a result, fails to identify secondary research identifying gene - outcome relationships. Many publications reference large amounts of retrospective molecular data derived from tissue banks and other sources. Some of these data are accessible in repositories like the genomic data commons and NCBI-GEO and would make an excellent addition to analyses performed for this study.

## Conclusion

There exists a rich set of currently inaccessible but normalizable data in biomarker-based clinical trial publications. In a subset of 13 mCRPC clinical trial publications reporting the results of trials measuring homologous recombination and DNA repair deficiency biomarkers, our approach demonstrates that biomarker clinical trials have sufficient normalizable data. This will help inform biomarker selection in clinical trial design, compare and rank survival metrics, investigate the effects of biomarker-outcome relationships, evaluate adverse events, and automate the discovery of therapeutic mechanisms. Today, oncologists, regulatory agencies, and researchers use many of these same data points, but they must search, read, and recall data from publications to retrieve them. The approach presented in this work to collect and store data allows for data to be reused, combined with other data sets, and extended in future analyses. While we showed its utility for metastatic prostate cancer, our approach is domain-agnostic and can be easily extended to other clinical domains and advance precision medicine.

## Data availability statement

The datasets presented in this study can be found in online repositories. The names of the repository/repositories and accession number(s) can be found below: https://insilica.co/bawto/; https://github.com/insilica/bawto.

## Author contributions

This project was directed by CP, with critical review, editing and feedback from LW. RG provided editorial and administrative support. Technical development and analysis was performed by TL. Project administration was provided by TB. All authors contributed to the article and approved the submitted version.

## Conflict of interest

Authors TL and TB were employed by Insilica LLC. The remaining authors declare that the research was conducted in the absence of any commercial or financial relationships that could be construed as a potential conflict of interest.

## Publisher's note

All claims expressed in this article are solely those of the authors and do not necessarily represent those of their affiliated organizations, or those of the publisher, the editors and the reviewers. Any product that may be evaluated in this article, or claim that may be made by its manufacturer, is not guaranteed or endorsed by the publisher.
