# Supplementary material for: Applications for open access normalized synthesis in metastatic prostate cancer trials
Source: Front Artif Intell. 2022 Sep 12;5:984836. doi: 10.3389/frai.2022.984836 (PMC9511148; doi:10.3389/frai.2022.984836)
Supplement: Supplementary file 1 [file Data_Sheet_1.docx]

# **Appendix**

**Glossary:**AAP Abiraterone Acetate + Prednisone
AE adverse event
AR androgen receptor
ARD androgen receptor directed
BAWTO biomarkers associated with therapeutic outcomes
DRD DNA repair deficiency
ENZ enzalutamide
HR hazard ratio
HRD: homologous recombination deficiency
PARP poly ADP ribose polymerase
PARPi poly ADP ribose polymerase inhibitor
PFS progression-free survival
PSA prostate specific antigen
mCRPC metastatic castration-resistant prostate cancer
ML machine learning
MOB model-based recursive partitioning
NLP natural language processing
OS overall survivability
RECIST response evaluation criteria in solid tumors
SER systematic evidence review
SLR systematic literature review


homologous recombination and DNA repair deficiency (HRD/DRD)

**A1 Clinical Trial Hazards -** Clinicaltrials.gov and AACT provide some programmatic access to adverse events from clinical trials. While Clinicaltrials.gov covered fewer trials but had more adverse events per trial. Clinicaltrials.gov also does a better job of normalizing vocabularies and has an organ/vocabulary system for reporting adverse events. It is possible that some of the adverse events captured in our research have yet to be added to the Clinicaltrials.gov system. In the below figure, adverse events are reported at the organ level in a format similar to Fig 6.

##
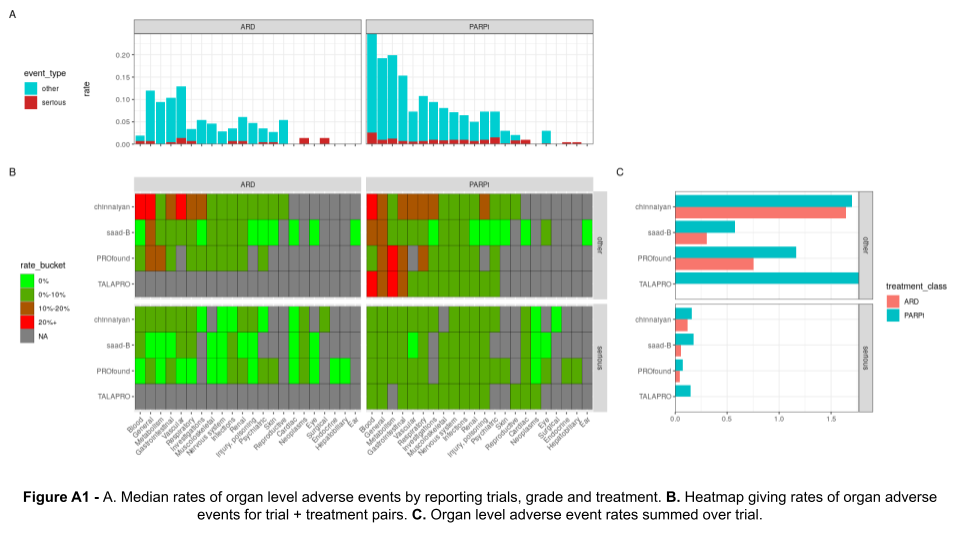


**A2 AAP vs. PARPi Risk Difference -** Reported adverse event rates in trials with both a PARP-i and AAP or Enzalutamide (ARD) arm can be used to evaluate risk differences. The below figure demonstrates some subtlety in risk differences at the low-grade level. At the high-grade level, anemia was the only adverse event that reached significance.
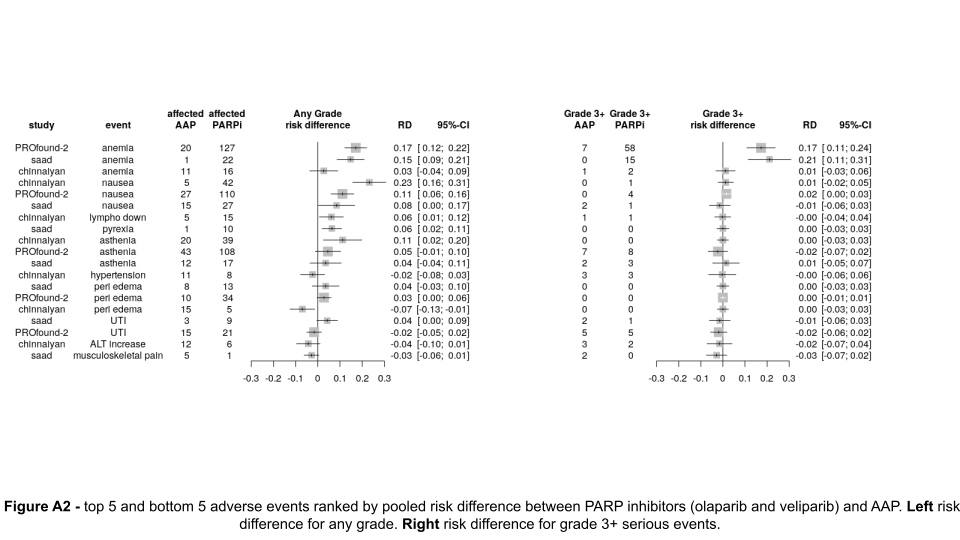


## 
